# A Collaborative Tale of Diagnosing and Treating Chronic Pulmonary Aspergillosis, from the Perspectives of Clinical Microbiologists, Surgical Pathologists, and Infectious Disease Clinicians

**DOI:** 10.3390/jof6030106

**Published:** 2020-07-11

**Authors:** Paige M. K. Larkin, Ashrit Multani, Omer E. Beaird, Ayrton J. Dayo, Gregory A. Fishbein, Shangxin Yang

**Affiliations:** 1 Department of Pathology and Laboratory Medicine, University of California Los Angeles, Los Angeles, CA 90095, USA; plarkin@northshore.org (P.M.K.L.); adayo@mednet.ucla.edu (A.J.D.); gfishbein@mednet.ucla.edu (G.A.F.); 2 Department of Pathology, NorthShore University HealthSystem, Evanston, IL 60201, USA; 3 Department of Medicine, Division of Infectious Diseases, University of California Los Angeles, Los Angeles, CA 90095, USA; amultani@mednet.ucla.edu (A.M.); obeaird@mednet.ucla.edu (O.E.B.)

**Keywords:** chronic pulmonary aspergillosis, *Aspergillus*, antifungal agents

## Abstract

Chronic pulmonary aspergillosis (CPA) refers to a spectrum of *Aspergillus*-mediated disease that is associated with high morbidity and mortality, with its true prevalence vastly underestimated. The diagnosis of CPA includes characteristic radiographical findings in conjunction with persistent and systemic symptoms present for at least three months, and evidence of *Aspergillus* infection. Traditionally, *Aspergillus* infection has been confirmed through histopathology and microbiological studies, including fungal culture and serology, but these methodologies have limitations that are discussed in this review. The treatment of CPA requires an individualized approach and consideration of both medical and surgical options. Most *Aspergillus* species are considered susceptible to mold-active triazoles, echinocandins, and amphotericin B; however, antifungal resistance is emerging and well documented, demonstrating the need for novel therapies and antifungal susceptibility testing that correlates with clinical response. Here, we describe the clinical presentation, diagnosis, and treatment of CPA, with an emphasis on the strengths and pitfalls of diagnostic and treatment approaches, as well as future directions, including whole genome sequencing and metagenomic sequencing. The advancement of molecular technology enables rapid and precise species level identification, and the determination of molecular mechanisms of resistance, bridging the clinical infectious disease, anatomical pathology, microbiology, and molecular biology disciplines.

## 1. Introduction

*Aspergillus* is a genus of environmental filamentous hyaline mold involved in invasive, chronic, and allergic disease [1]. *A. fumigatus*, *A. flavus*, *A. niger*, *A. terreus*, *A. clavatus*, and *A. nidulans* are most frequently implicated in invasive aspergillosis, with *A. fumigatus* being the most common [1,2,3]. *Aspergillus* spp. are found in the soil and air, and humans inhale an estimated 100–1000 spores per day [1]. An immunocompromised state is a risk factor for severe disease [1].

Chronic pulmonary aspergillosis (CPA) is composed of a spectrum of *Aspergillus*-mediated disease, including chronic cavitary pulmonary aspergillosis (CCPA) and subacute invasive pulmonary aspergillosis (SAIA) (AKA chronic necrotizing pulmonary aspergillosis, CPNA) [4,5]. While its incidence is likely vastly underestimated [4,5], CPA is associated with high morbidity and mortality [2,6]. The risk factors include chronic obstructive pulmonary disease (COPD), allergic bronchopulmonary aspergillosis, sarcoidosis, and prior mycobacterial infection [4,7,8]. More than three months of persistent symptoms of chronic inflammation, including chronic productive cough, weight loss, fatigue, mild hemoptysis, and dyspnea, are key diagnostic criteria [4,5]. 

The combination of culture, histopathology, fungal serology, clinical symptoms, and radiographic features is typically used for the diagnosis of CPA [4]. Histopathologic findings include chronic inflammatory infiltrations, fibrosis, and cavitation [2]. Classically, the organisms appear as septate hyphae with acute angle branching [2]. However, currently, only culture can provide species-level identification, which is essential for antifungal susceptibility. Resistance to azoles, one of the first line treatments for *Aspergillus* spp. [7], is increasing in prevalence in *Aspergillus fumigatus* [9,10]. Triazole resistance has also been documented in certain cryptic *Aspergillus* spp. [11], emphasizing the importance of species-level identification in guiding treatment. With only half of fungal cultures being positive for patients with CPA [4], new molecular techniques are being developed to improve diagnostics.

Here, we provide an in-depth discussion on clinical presentation, and the strengths and pitfalls of current and future diagnostics and treatments for CPA. With the advancement of molecular technology, precise species level identification and molecular mechanisms of resistance can be achieved, bridging the clinical infectious disease, anatomic pathology, microbiology, and molecular biology disciplines. 

## 2. Clinical Presentation and Definition 

### 2.1. Different Forms of Chronic Pulmonary Aspergillosis, Risk Factors, and Diagnosis 

The term CPA refers to several disease entities. While tissue destruction is a component in more severe forms, CPA is considered distinct from acute invasive pulmonary aspergillosis (IPA), which typically occurs in individuals with substantial immunodeficiency, such as in those with prolonged neutropenia due to hematologic malignancy, and solid organ or stem-cell transplant recipients [5]. CPA generally occurs in the setting of predisposing anatomical and structural abnormalities, such as old cavitary lesions, and does not require significant host immunosuppression to develop, although mild or moderate host immune dysfunction likely contributes to pathogenesis in some cases [6,12]. The spectrum of disease ranges from the asymptomatic colonization of existing pulmonary cavities, as in simple aspergillomas, to chronic and progressive pulmonary disease, as seen in chronic cavitary pulmonary aspergillosis (CCPA) and chronic fibrosing pulmonary aspergillosis (CFPA) (Table 1). Subacute invasive (i.e., chronic necrotizing) pulmonary aspergillosis (SAIA/CNPA) falls between IPA and the other forms of CPA [12], and will be discussed here.

Structural lung abnormalities are present in the majority of patients with CPA. Prior tuberculosis (TB) is the most common underlying condition, followed by history of non-tuberculous mycobacterial (NTM) lung disease; other pre-disposing conditions include allergic bronchopulmonary aspergillosis (ABPA), fibrocavitary sarcoidosis, COPD, asthma, and a history of lung cancer [6,8]. Most patients with CPA outside of those with SAIA are not overtly immunosuppressed, however there is some literature suggesting that genetic defects affecting the innate immune response may predispose individuals to CPA [12]. 

An aspergilloma is a fungal ball consisting of *Aspergillus* hyphae, and the associated extracellular matrix [12]. Aspergillomas are characteristic and can be seen in all types of CPA, with the exception of nodular disease [12]. Simple aspergilloma refers to a single pulmonary cavity containing a fungal ball, that remains radiographically stable over at least three months [12]. Patients with simple aspergillomas typically lack any pulmonary or systemic symptoms [12]. 

CCPA is the most common form of chronic aspergillosis. Radiographically, this form of disease is characterized by the presence of multiple cavities, with or without aspergillomas [8]. The disease is characterized by slow progression with the formation of new cavities or coalescence of existing cavities with pericavitary infiltrates and/or pleural thickening [12]. Patients have significant chronic pulmonary and systemic complaints, including weight loss, cough, dyspnea, hemoptysis, and fatigue [5,8,12]. Fever and night sweats are less common [8]. Symptoms are chronic, occurring over months to years [5]. Untreated, CCPA can progress to CFPA, which is characterized by extensive fibrotic destruction [5,13]. This results in a substantial loss of lung function [12]. 

*Aspergillus* nodules are uncommon in CPA, but when present, are typically non-cavitary unless large, and can be solitary or multiple [12]; radiographically, they are difficult to distinguish from other nodule forming pulmonary diseases, such as endemic fungal infections, including coccidioidomycosis and chronic cavitary histoplasmosis, cryptococcosis, non-tuberculous and tuberculous mycobacterial infection and neoplasm [12,13]. Patients are typically asymptomatic, although a cough may be present. 

SAIA occurs in individuals with underlying immune compromising conditions, such as elderly or malnourished patients, as well as those with poorly controlled diabetes mellitus, glucocorticoid use, and alcoholism [12,13]. The pathophysiology more closely resembles that seen in IPA, with the presence of tissue invasion by fungal hyphae. Clinical and radiographic manifestations resemble CCPA, however, this occurs over a course of weeks to months [12]. 

### 2.2. Brief Overview of the Collaborative Approach of Diagnosing CPA

Diagnostic criteria are noted in Table 1, highlighting the multidisciplinary approach for diagnosis. Diagnosis is made on the basis of the compatible clinical, radiographical and microbiological or serological evidence of *Aspergillus* infection, or by histology. This collaborative approach will be described in the following sections. 

For clinical diagnosis, by definition, the disease has to be present for at least three months, based on current diagnostic criteria [13]. The symptoms of CPA are not distinguishable from other chronic respiratory infections; the most common symptoms being weight loss (94%) and chronic cough (78%), and more variably, hemoptysis (58%) and dyspnea (50%), with fevers being uncommon (11%) [5]. CPA should be considered in a patient presenting with these complaints, particularly if they have a known history of underlying structural lung disease. The clinical presentation of CCPA closely resembles TB and NTM infection, which can occur concurrently [12]; evaluation for these, as well as the exclusion of endemic fungal infection, is important, particularly in patients with an appropriate history of geographical exposure [8,13]. Acute bacterial superinfection of cavitary lesions is possible and should be considered in the presence of fevers [8]. 

As noted in Table 1, radiographic features can help one to discern between the different subtypes of CPA, and when used in conjunction with clinical history and laboratory testing, they are required for diagnosis. Chest x-ray is often the first modality used, given its ease and availability. However, the computed tomography (CT) of the thorax provides more fidelity, including the better visualization of the distribution and extent of lung involvement. The presence of an aspergilloma within a cavitary lesion is suggestive of CPA, however, it is not seen in all cases [13]. By definition, CPA is characterized by change over time, and serial imaging is required in order to demonstrate progression, or lack thereof. 

If CPA is suspected, serum *Aspergillus* IgG should be ordered, as this is the most sensitive serological test in CPA [8,13]. Antibody testing for *Aspergillus* precipitins appears to have lower sensitivity than conventional *Aspergillus*-specific IgG assays [14]. If antibody testing is negative, other studies to evaluate for the presence of *Aspergillus* should be performed, including *Aspergillus* antigen, PCR or fungal cultures from respiratory specimens or excisional biopsy [13]. Caution should be taken with the interpretation of positive sputum cultures or PCR in isolation, given the potential for airway colonization. However, in the appropriate clinical context, these tests can be useful in confirming a diagnosis of CPA [13]. The strengths and weaknesses of histopathology and microbiological studies will be further explored in the following sections.

## 3. Histopathology

In the lung, there is a diverse spectrum of pathologic findings associated with *Aspergillus* spp. Host factors, such as hypersensitivity and immunocompetency, are important parameters that largely dictate the pathologic findings. In essence, the histopathology of *Aspergillus* can be divided into three categories: colonization, hypersensitivity reaction, and invasive infection. Very commonly, inhalational exposure to *Aspergillus* spp. is sufficient to cause disease in hypersensitive individuals. Malt-worker’s lung and Tobacco-worker’s lung are examples of hypersensitivity pneumonitis associated with exposure to *Aspergillus*, in the form of moldy barley and tobacco mold, respectively [15]. In these examples, the organisms will not be identified histologically, and there is no role for special stains. 

**Figure 1:**
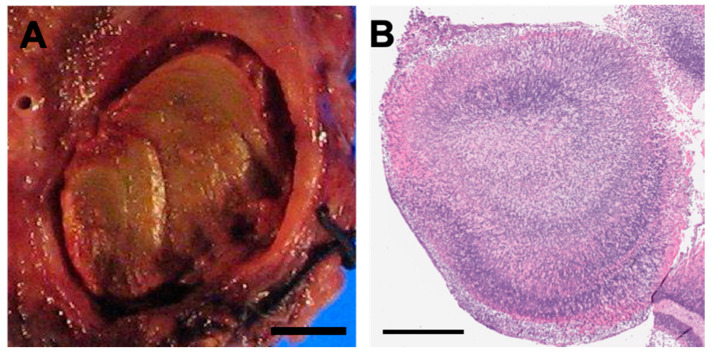
Aspergilloma Pathology and Histopathology. (**A**) Gross photograph of aspergilloma (aka fungus ball) in a colonized patient with bronchiectasis. The ball is inhabiting a cystic cavity; no invasion of the lung tissue is present (bar = 0.5 cm). (**B**) H&E stained section of a fungus ball. Not the variable basophilic to eosinophilic staining and arboreal growth pattern (bar = 500 microns).

**Figure 2:**
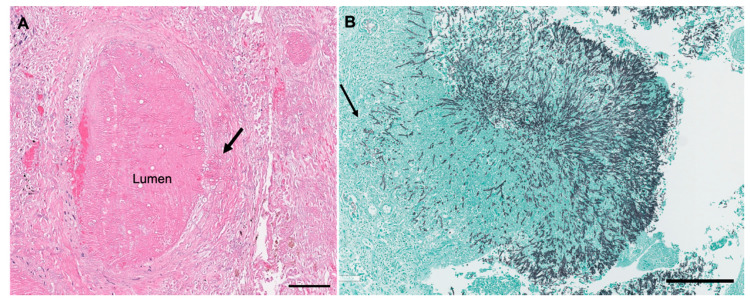
Angioinvasive and Superficially Invasive *Aspergillus* spp. (**A**) A focus of angioinvasion in invasive aspergillosis. The hyphae invades the wall of the blood vessel (arrow) and into the lumen. The surrounding tissue is necrotic (H&E). The scale bar represents 200 microns. (**B**) Fungus ball in bronchiectatic cavity with superficial invasion into the airway wall (GMS). The scale bar represents 300 microns.

In contrast, colonization of the fungus in the lower respiratory tract may result in a distinct hypersensitivity reaction, allergic bronchopulmonary aspergillosis, in which the main pathologic findings are asthma, mucoid plugging with allergic mucin, bronchocentric granulomatosis, and eosinophilic pneumonia [16]. Colonizers with pre-existing airway obstruction or cavitary lesions may develop aspergillomas, also called fungus balls (Figure 1). In most immunocompetent individuals, the fungal ball will merely “coexist” with the pre-existing condition, and will not invade the lung parenchyma. However, mildly immunocompromised individuals may develop SAIA/CNPA, sometimes considered a “semi-invasive” pattern of infection. Well-formed fungus balls may be present, but with varying degrees of infiltration and destruction of the adjacent airway and pulmonary parenchyma. The reaction is usually granulomatous, either in the form of necrotizing granulomatous pneumonia, granulomatous bronchiectatic cavity, or granulomatous bronchiolitis [17]. More significantly immunocompromised patients may develop IPA. Pathologically, CNPA is distinguished from IPA by the limited extent of parenchymal infiltration and the absence of vascular invasion and infarction. In IPA, the vascular invasion can lead to widespread dissemination out of the lung and into the distant organs. Invasive and superficially invasive pulmonary aspergillosis can be seen in Figure 2.

**Figure 3:**
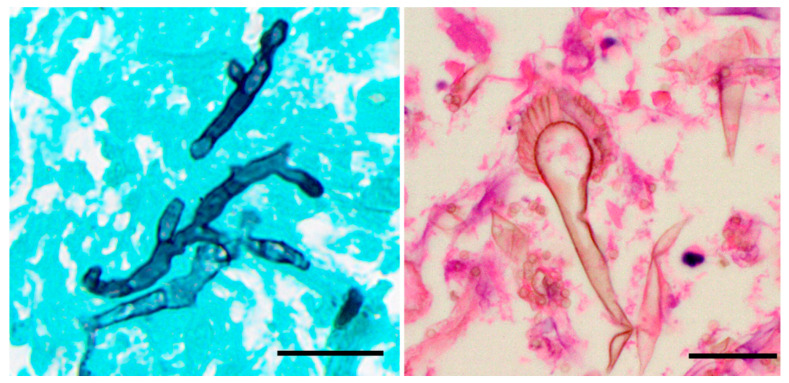
*Aspergillus* spp. Histopathology. (**A**) Grocott–Gomori’s methenamine silver (GMS) stain, demonstrating septate hyphae with acute angle branching. (**B**) Conidial heads composed of conidiophores, with a terminal vesicle and one or two layers of phialides may be seen, usually in cavitary lesions (H&E). The scale bar is 20 microns for both images.

In histologic sections, the well-preserved organisms of *Aspergillus* spp. appear as uniform septate hyphae measuring 3–6 µm in width, but may be larger when degenerated (up to 15 µm) [18,19]. They have regular septation, parallel contours, and a progressive arboreal pattern of branching. Branches are dichotomous and predominantly arise at acute (45°) angles [18]. The hyphae are visible on hematoxylin and eosin (H&E)-stained sections, but show variable staining. Viable hyphae have basophilic walls, but necrotic organisms may appear to be eosinophilic or colorless. Conidial heads are rarely produced within human tissues, but may be seen when the fungi are exposed to air, such as in cavitary lesions (Figure 3). Grocott–Gomori’s methenamine silver (GMS) or periodic acid Schiff (PAS) stains are extremely helpful in identifying the organisms [20,21].

**Figure 4:**
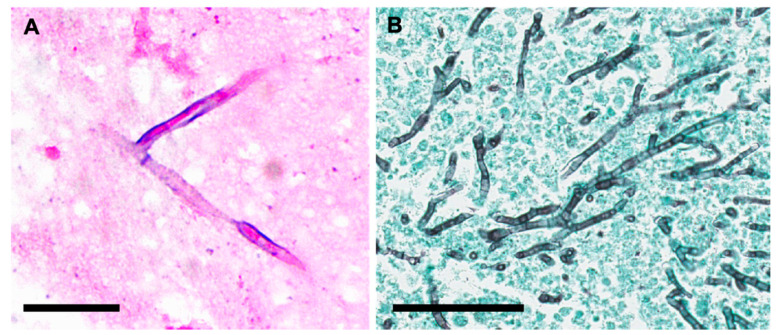
Unusual Morphology and Morphological Mimics. (**A**) *Aspergillus fumigatus*, demonstrating apparent right-angle branching. This could easily be confused with *Fusarium* spp. (PAS; bar = 20 microns). (**B**) Septate branching hyphae of *Aureobasidium nambiae* in a necrotizing granuloma in the lung; note the acute-angle branching. The hyphae appear to be virtually identical to *Aspergillus* spp. (GMS; bar = 70 microns).

It may be difficult or impossible to distinguish *Aspergillus* form other organisms that demonstrate septate branching hyphae (Figure 4). One study of 122 specimens found that in 17% of cases with filamentous fungi, the histology and cultures yielded discordant results [22]. In the discordant cases, cultures grew *Scedosporium*, *Fusarium*, *Phialophora verrucosa*, and *Trichophyton* [22]. In addition, the degenerated hyphae of *Aspergillus* may demonstrate globose segments up to 15 µm in width, with inconspicuous septa and sparse branching; these forms may closely mimic the hyphae of Mucorales [19]. Therefore, unless the typical conidial heads are present, a definite diagnosis of *Aspergillus* based on histopathology alone is likely unreliable; the diagnosis is best confirmed by additional microbiology studies. Despite this limitation, histopathology remains the gold standard for distinguishing invasive from non-invasive disease.

## 4. Culture-Based Microbiological Methods for Identification and Antifungal Susceptibility Testing

### 4.1. Morphological Identification

The growth of *Aspergillus* from fungal culture is supportive of a diagnosis of CPA in the correct clinical context. A positive culture in isolation does not confirm disease, owing to the ubiquitous nature of *Aspergillus* and potential of airway colonization and/or laboratory contamination. Lower respiratory tract specimens such as bronchoalveolar lavage (BAL) are preferred, but sputum is considered acceptable [7]. An estimated 11.8–81.0% of respiratory specimens [23] and only half of sputum from patients with CPA are positive by fungal culture [4], but the positive rate can be improved by submission of multiple specimens and higher volumes [7]. It is important to note that false positive culture results can occur due to airway colonization and contamination [23]. When examining fungal growth, a list of observations may be noted to determine contamination, including specimen type, quantity and placement of growth on culture, pattern of growth, and number of cultures growing the same organism. Other pertinent patient information, such as previously recovered organisms and antifungals being administered to the patient that could distort classic mold morphology, can also aid in distinguishing laboratory contaminants. In conjunction with culture, direct stains performed in microbiology, such as calcofluor stain, can be helpful in identifying true infection, and provide early information regarding the need for treatment. 

In order to maximize the recovery of *Aspergillus* in culture, proper specimen collection and transport are of paramount importance. Long delays in transport lower the recovery rate of medically important fungi, as it allows the specimen to become overgrown with bacteria and saprophytes [24] (p. 365). *Aspergillus* spp. generally appear in culture within the first two days of incubation and mature by the third day [24,25] (p. 293, p. 56). Identification, however, may take longer depending on the organism morphology on primary isolation and the presence of resistant, contaminating bacteria. 

**Figure 5:**
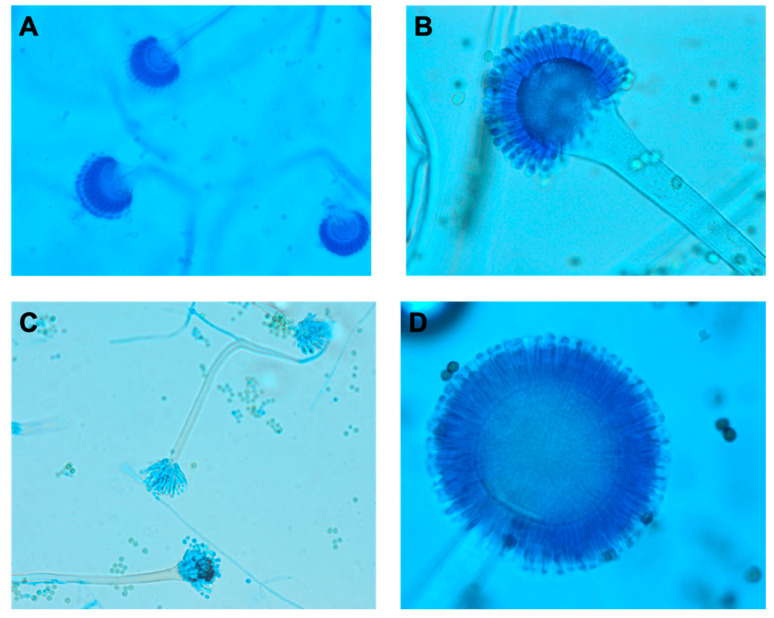
Microscopic Morphology of *Aspergillus* spp from Fungal Culture. Lactophenol blue was used to prepare slides for the following *Aspergillus* spp: (**A**) *Aspergillus fumigatus*, (**B**) *Aspergillus flavus*, (**C**) *Aspergillus nidulans*, (**D**) *Aspergillus niger*. The rough pitted stalks distinguish *A. flavus* from other *Aspergillus* spp, while the black conidia of *A. niger* sets it apart. Table 2 goes into further details regarding key microscopic and morphological differences between *Aspergillus* spp.

Traditionally, the species identification of *Aspergillus* is based on a combination of macroscopic and microscopic morphology [3]. Microscopic identification entails the characterization of the morphology of the conidia (spore) and conidophores (Figure 5). Microscopic examination is best performed on younger cultures, when organisms are most actively sporulating, to visualize their characteristic structures [24] (p. 372). In general, *Aspergillus* produces septate and hyaline hyphae. Key factors that can be used to differentiate the most commonly isolated species of *Aspergillus* can be found in Table 2 [24,25] (p. 293, p. 56). *A. fumigatus*, the most common cause of CPA, exhibits chains of green echinulate conidia in the absence of metulae [26].

### 4.2. Mass Spectrometry

Matrix-assisted laser desorption ionization time-of-flight mass spectrometry (MALDI-TOF MS), for mold identification, offers an advantage over morphological identification. One multicenter study found that 91% of fungal isolates were correctly identified to the species level and an additional 2% of isolates were correctly identified to the genus level by MALDI-TOF MS [27]. In this study, the accuracy for *Aspergillus* species level identification ranged from 71-100%, with *Aspergillus versicolor* being the most problematic [27]. In another study, MALDI-TOF MS was shown to correctly identify *A. fumigatus*, *A. lentulus*, *A. niger*, *A. tubigenesis*, *A. nidulans*, *A. sydowii*, *A. unguis*, *A. terreus*, and *A. alabamensis* to the species level; in addition, culture media, incubation temperature, or the pretreatment of molds did not impact the accuracy [28]. 

However, there are important pitfalls to consider. There is variability in the results across institutions and databases [29], with species level identification ranging from 32–77% across eight testing centers [30]. This was partly attributed to the lack of standardization of instrument settings, including key spectral acquisition and processing parameters [30]. The age of fungal culture can also impact the results, likely due to differences in sporulation [28]. Misidentification and no identifications occur as a result in gaps and differences in reference databases [27,31,32]. This has led to the misidentification of *A. flavus*, *A. nomius*, and *A. tamarrii* by MALDI-TOF MS [33]. To further improve the accuracy, some laboratories have extended their databases through the implementation of laboratory-developed databases [32], or have implemented threshold and validation criteria to improve results [34]. One study found 100% concordance between sequencing and MALDI-TOF MS species-level results for *Aspergillus* spp, using an in-house developed reference database [35]. Due to these limitations, the utilization of MALDI-TOF MS for mold identification is not yet widespread.

### 4.3. Antifungal Susceptibility Testing

Across *Aspergillus*-mediated diseases, including CPA, there is a rise in azole resistance, particularly in European countries [7,9,10,11,36]. Thus, susceptibility testing is becoming more common for guiding the treatment of *Aspergillus* spp in CPA, especially *A. fumigatus*. The true prevalence of azole-resistant *Aspergillus* varies across countries and hospital systems and is largely unknown for CPA and other *Aspergillus*-mediated diseases; therefore, the routine surveillance of *Aspergillus fumigatus* isolates for azole-resistance is recommended [36]. Moreover, cross resistance across azoles is a growing concern, with one study in the United Kingdom showing that 65% and 74% of itraconazole-resistant *A. fumigatus* clinical isolates were cross resistant to voriconazole and posaconazole, respectively [37], further limiting the effective treatment options. These clinical isolates included one or more *A. fumigatus* isolates from CCPA and non-CCPA patients [37]. Cross resistance to azoles in CPA has been documented in other studies, including one case report describing a CPA patient, where multiple pan-azole resistant *Aspergillus fumigatus* isolates were recovered [38]. Inducible azole-resistance is a concern and should be monitored with prolonged therapy. Prior azole therapy was administered 1–30 months before the identification of the 1st resistant isolate in a study that included multiple CCPA patients [37].

However, antifungal susceptibility testing remains a controversial practice. The correlation between *in vitro* antifungal susceptibility testing and clinical response is less clear, and thus the ordering of antifungal susceptibility testing is up to the discretion of the clinician and is not yet routine, except in the case of suspected resistance [39,40,41]. 

Susceptibility testing for filamentous fungi can be performed using broth dilution methodology, following the standards published by Clinical and Laboratory Standards Institute (CLSI) or European Committee on Antimicrobial Susceptibility Testing (EUCAST) [42]. Fungal susceptibility testing is not routinely performed in clinical laboratories, due to labor and training requirements [42], limiting the availability of such testing. Alternative assays have been evaluated for *Aspergillus*, including the use of the Sensititre YeastOne Method (Thermo Fisher Scientific) [43,44] and an agar-based Etest MIC [45]. 

## 5. *Aspergillus* Serology

For diagnosing CPA, several serological assays are commonly used: 1,3-Beta-D-glucan (BDG, Fungitell), galactomannan (GM, *Platelia Aspergillus*) and *Aspergillus fumigatus* immunoglobulin G (IgG) and IgE antibody.

*Aspergillus fumigatus* immunoglobulin G (IgG) antibody is positive in the vast majority of patients with CPA [13]. *Aspergillus fumigatus* IgG titers can also be used to track response to therapy, although titers do not tend to become negative over time [8,13]. For proven CPA, *Aspergillus* IgG was positive in 97.9% of cases in one study [46]. Proven CPA cases had a significantly higher *Aspergillus* IgG level compared to the control group when using a quantitative assay [46], supporting the utility of *Aspergillus* IgG in CPA diagnosis. While a positive *Aspergillus fumigatus* IgG result has a high positive predictive value in CPA, positive results can also be seen in other conditions, including ABPA and IPA [47,48]. Furthermore, there is substantial variability in the currently available methods, and a lack of standardization in testing. Additionally, while the majority of cases of CPA are caused by *Aspergillus fumigatus*, serologic testing for non-fumigatus species is not widely available, although the literature suggests cross-reactive IgG with other *Aspergillus* spp [49]. Other potential lab abnormalities can include elevated total IgE, *Aspergillus fumigatus* specific IgE, and elevations in inflammatory markers C-reactive protein or erythrocyte sedimentation rate (ESR), although these are non-specific. *Aspergillus fumigatus* IgE test (SorB and QoEII) is recommended in the asthmatic and cystic fibrosis patient populations [13].

With regards to BDG and GM assays, while they are routinely used in diagnosis due to the non-invasive nature of the assays, cross reactivity and sensitivity are prominent issues with these assays [18,50]. BDG is found in the cell wall of many fungi, including *Aspergillus* and *Candida* spp, making it a useful test for invasive fungal infections, but not a specific test for *Aspergillus*. Both serum and BAL can be tested, although BAL has lower specificity and can have problematic reproducibility compared to serum [51]. While sensitivity and specificity varied according to the patient population [52], in a multicenter study, BDG assay exhibited 77.8% sensitivity across patient populations testing in serum, with IPA having the lowest sensitivity (68%) [53]. BDG sensitivity and specificity was 77.8% and 72.5%, respectively, in BAL samples from CPA patients [54]. False positive BDG assays have been attributed to gram-negative infections [52], bacteremia [55], the infusion of polyclonal IgM-enriched immunoglobulins [52], the *Candida* spp. colonization of a respiratory source [56], and β-lactam antibiotics [57], while false negative results could be due to antifungal use [51]. 

GM is a polysaccharide component of the fungal cell wall that is present in detectable levels in serum when there is tissue and/or angioinvasion [58,59]. This antigen is detected using a sandwich enzyme-linked immunosorbent assay [58]. To establish a true positive result in serum, the use of a cutoff of GM index ≥ 0.5 based on two separate serum samples or ≥1.0 in a single serum sample is recommended [59]. The utility of serum GM is well established in IPA, however this test generally has poor sensitivity in CPA with the exception of SAIA. Although GM is more specific for *Aspergillus* than BDG, exoantigens from other molds, including *Acremonium*, *Penicillium*, and *Fusarium* can cross react [58]. Sensitivity ranges from 13–100%, depending on the patient population, specimen source, use of antifungal treatment, *Aspergillus* spp., and institution [58,60]. The testing of GM from BAL specimens has increased sensitivity, with reported rates of 85.7% and 92% for aspergilloma and SAIA, respectively, using a cut-off optical density of 0.5 or greater, with specificity of 76.3% and 78.9%, respectively [12,13], while the sensitivity and specificity of GM in BAL for CPA were found to be 77.8% and 90.0%, respectively, in one study [54]. An important consideration is the increased rate of false positives observed in neonatal and pediatric patients compared to adults [61,62]. Other false positives have been documented for *Bifidobacterium bifidum* [63], exoantigens from other molds [58], piperacillin-tazobactam treatment [64], intravenous human immunoglobulin administration [65], amoxicillin-clavulanic acid treatment [66], *Cryptococcus neoformans* infection [67], and β-lactam antibiotics [57]. Specificity can be improved by requiring more than one positive GM test result to be considered clinically relevant [58].

When comparing the two assays, the use of GM in serum samples was found to be more specific [57], but less sensitive than BDG, for cases of aspergillosis [51,53,57,60]. Bacteremia is a significant factor for causing the false positive results for BDG, but has less influence for GM results [57]. When specifically comparing the use of BDG and GM on BAL for patients with CPA, sensitivity was comparable between the assays [54]. With these strengths and limitations, there is not a clearly favored serological method for CPA, and thus, the use of these assays varies across institutions [51,68]. Because of these limitations, serology should be used to support a diagnosis of CPA in the presence of compatible clinical and radiographical features, but alone cannot establish a diagnosis. 

## 6. Molecular Methodologies for *Aspergillus* Diagnosis and Antifungal Susceptibility Predictions 

### 6.1. Aspergillus PCR

Although not yet routinely performed for CPA, PCR has been suggested to supplement serological testing to improve sensitivity [69], and can be used to rule in invasive *Aspergillus* in patients with consistent signs and symptoms [70]. Common PCR targets include 28S rRNA gene, internal transcribed spacers (ITS), and 18s rRNA gene [71], and assays can be singleplex or multiplex [71]. Multiplex PCRs are desirable, as they can detect multiple clinically relevant *Aspergillus* spp. However, sensitivity and amplification efficiency across targets are two major concerns with multiplex PCR for *Aspergillus* [72]. False positivity due to residual or transient *Aspergillus* DNA is also a concern [72]. Respiratory sources are more likely to have environmental contamination than sterile sites, such as peripheral blood [72]. 

There have been efforts to standardize *Aspergillus* PCR, including the formation of the European *Aspergillus* PCR Initiative [73], which has provided recommendations for *A. fumigatus* PCR in whole blood, serum, and plasma [71]. *A. fumigatus* and *A. flavus* mitochondrial DNA has been successfully detected in serum using a lab-developed PCR test, although with a lower positive rate compared to the GM assay [74]. A comparison of an *Aspergillus* PCR on whole blood and serum demonstrated a sensitivity of 85.1% and 78.7%, respectively, but serological assays can be performed on the same serum specimen, making it a more attractive option [69]. Other studies demonstrated the sensitivity of PCR assays for detection of *Aspergillus* in BAL specimens as 44–86.7%, depending on different protocols [54,75]. In CPA patients, the sensitivity and specificity of two *Aspergillus* PCR assays performed on BAL ranged from 66.7–86.7% and 84.2–94.2%, respectively [54]. It is critical to note that insufficient specimen volumes or samples taken from patients with antifungal treatment can have a reduced fungal burden that is below the limit of detection of the PCR test, resulting in a false negative result, and thus, this should be interpreted with caution [70].

### 6.2. Sequencing

Pan-fungal PCR, followed by Sanger sequencing assays, have been available for more than a decade [76,77,78,79], with movement towards targeted next-generation sequencing (NGS) assays following pan-fungal PCR. The use of such technologies requires primers that target conserved regions of the fungal genome, that also exhibit enough diversity to differentiate between species of fungi. Ideally, the primers should be designed to promote amplification the sequences of all clinically relevant fungi. Widely used targets include ITS and the D1–D2 region of the large subunit of 28S rRNA gene. The ITS region is hypothesized to cover the broadest spectrum of fungi [80], while the ITS2 region (using ITS 3–4 primers) achieved the best identification for the common clinically significant yeasts and molds, including *Aspergillus* spp., *Blastomyces dermatitidis*, *Histoplasma capsulatum*, and *Rhizopus* spp. [78]. However, caution should be taken when providing species-level identification of *Aspergillus* spp., due to the sequence similarity across conserved regions. The percentage identity of *Aspergillus* spp. in ITS1, ITS2, and D1–D2 region were 57.4–98.1%, 75.6–98.3%, and 91.9–99.6%, respectively, across 13 clinically relevant *Aspergillus* spp. [81] Alternatively, sequencing multiple target genes, such as ITS, β-tubulin, and calmodulin genes, has been used for the most accurate species-level identification for *Aspergillus* spp. [28,33,35].

For CPA, the ideal sample for NGS is formalin-fixed paraffin-embedded (FFPE) tissues. As previously discussed, organism recovery in fungal culture from respiratory samples taken from CPA patients is low, with less than half of sputum samples growing a fungal organism [4]. Thus, organism identification falls on histopathology, which cannot provide species-level identification. While the initial histopathology analysis of the FFPE tissue allows for the assessment of fungal morphology, as well as a degree of inflammation and invasion, a targeted sequencing approach could then be used to accurately identify the *Aspergillus* species, guiding treatment and providing a more definitive diagnosis of the CPA in the absence of fruitful fungal culture. Moreover, an ongoing dilemma in infectious disease diagnostics is the limited specimen available for studies, which has to be further divided between microbiology and pathology, as the formalin used in pathology kills any viable fungi. This often leads to a lack of specimens submitted to microbiology for fungal culture workup. NGS can be performed directly on slices of FFPE tissue, allowing for the maximization of opportunities for organism identification. One limitation of FFPE tissue is that the degradation of fungal DNA negatively affects molecular testing [82]. However, fungal DNA has been recovered in FFPE blocks up to seven years after fixation [83]. Although protocols do exist with demonstrated success [78], commercial amplicon-targeted NGS assays are not yet available for FFPE.

Metagenomic sequencing can also utilize a shotgun approach for a “catch all” method. Shotgun metagenomics sequences all RNA or DNA within a sample, allowing for the detection of bacteria, fungi, parasites, and viruses through the taxonomic identification of the sequences [84]. This is particularly useful in settings where the differential diagnosis is not confined to one particular group of organisms. Commercially available sequencing is available for plasma [85], CSF sources [86], and respiratory sources [87], with the former utilizing a cell-free DNA sequencing approach. False negatives are demonstrated with both cell-free DNA methods [88] and shotgun metagenomics. One study found that *A. fumigatus* and *Rhizopus oryzae*, which were recovered by culture, were not identified by cell-free DNA sequencing [88]. Thus, negative results from any metagenomic method should be interpreted with caution.

One major downside to shotgun metagenomics is the interference with human DNA and environmental contaminants [89,90,91,92]. Specimen pre-processing can increase pathogen-to-human DNA ratio, allowing for increased pathogen detection sensitivity and reduced human sequences [93]. Importantly, false positive results due to the contamination of normal flora during sample collection, environmental contamination during sample processing and misaligned taxonomic calls during bioinformatics analysis are possible, and should be interpreted carefully. In addition, metagenomic techniques tend to detect the transient or contaminant DNA of microorganisms in human blood or body fluids, with unknown clinical relevance [84,94,95]. Therefore, it is recommended to have both clinical microbiologists and infectious disease physicians involved in the interpretation of metagenomic results. To date, there are not any published studies investigating the use of shotgun metagenomics, specifically in the CPA patient population. However, as metagenomics approaches increase in popularity, this is an avenue that will likely be further explored. 

With the increased sensitivity of sequencing assays, contamination and false positive metagenomic sequencing results must be evaluated carefully [91]. Moreover, sequencing detects DNA, which is not an indication of viability [96]. Therefore, it is recommended to have a clinical microbial sequencing board attended by experienced clinical microbiologists knowledgeable about sequencing based technologies, in conjunction with thorough communication with the treating physicians and pathologists, to guide the interpretation of these NGS results in a case-by-case approach [86]. Establishing stringent bioinformatic cut-offs can also reduce background noise, such as using a standard deviation above the mean read approach for defining significance [97]. Currently, one biggest limitation for the clinical application of microbial NGS testing is the lack of availability and standardizations [96,98]. Sequence databases can also be limited, especially in *Aspergillus* spp. [81] Regardless, the application of NGS in clinical microbiology laboratories is predicted to increase substantially as the technology becomes more accessible and standardized.

### 6.3. Whole Genome Sequencing (WGS)

Unlike NGS-based metagenomics, WGS requires a pure isolate from culture, but can provide more in-depth information, including antimicrobial resistance mechanisms, strain types, and genetic relatedness between isolates [96,98], which is crucial for guiding treatment and supporting infection prevention. The utility of WGS for fungal resistance and phylogenetic analysis has been well demonstrated in *Candida auris* [99,100,101]. 

WGS has been increasingly utilized in mycology for the sequencing of *Aspergillus* spp., including *A*. *niger* complex [102], *Aspergillus terreus* [103], and *Aspergillus fumigatus* in pulmonary aspergillosis and chronic necrotizing aspergillosis [104]. One of the greatest advantages of WGS is the ability to detect specific mutations in the *cyp51A* gene [105], which is associated with azole resistance in *A. fumigatus* [42,106,107]. This particular mutation, leucine 98 to histidine (L98H), in combination with a 34 base pair tandem repeat in the promoter of *cyp51A* gene, has been well documented in the literature [106,107,108], and thus, the WGS of *Aspergillus* spp. can potentially detect resistance genes and predict drug resistance to a first line therapy. Interestingly, the *in vitro* synergy observed for posaconazole and caspofungin was found to be dependent on the genetic azole resistance mechanism found in varying *A. fumigatus* strains [109], highlighting the need for WGS to best tailor treatment. Moreover, acquired resistance mutations over time can be determined using WGS, as was found for sequential *Aspergillus* spp. isolates from CPA patients [110]. In this study, multiple punctual mutations, including a mutation in *cyp51A*, and a large segment deletion, was observed among *A. fumigatus* strains, supporting genomic rearrangement during infection [110]. Thus, WGS provides a mechanism for understanding acquired genetic resistance throughout treatment and disease course.

While there are ongoing efforts to develop pipelines to detect resistance genes by the identification of secondary metabolic gene clusters harboring potential resistance genes [111], it is important to also note the limitations of WGS. First, a pure isolate is required, and thus, this cannot be performed directly from the clinical specimen. Second, the quality of sequencing, criteria used for variant calling, assembling process, and choice of reference genome can greatly vary results, especially with single nucleotide variant (SNV) calling [112]. One study found that *A. fumigatus* reference genomes belonged to difference clusters and led to differences in SNVs calling [112]. Choosing the most closely related reference genome is important.

## 7. Treatment

### 7.1. Indications

The treatment of CPA requires an individualized approach and the consideration of both medical and surgical options. Treatment is not required for asymptomatic patients with serological and radiological stability [8,13]. Such patients can be managed expectantly with close follow-up every 3–6 months [8,13]. Treatment is indicated for patients with clinical, serological, and radiological evidence of progressive disease [8,13]. 

### 7.2. Role of Species Identification and In Vitro Antifungal Susceptibility Testing

Most *Aspergillus* species are considered to be susceptible to mold-active triazoles, echinocandins, and amphotericin B. However, certain species, such as *A. terreus*, *A. nidulans*, and *A. lentulus*, are considered to be resistant to amphotericin B, and *A. ustus* is considered resistant to multiple antifungals [8,113,114]. In the absence of species-level identification, empirical individualized antifungal therapy should be chosen, with close monitoring for clinical, serological, and radiological treatment response. *In vitro* antifungal susceptibility testing has not been proven to correlate with clinical outcomes in the treatment of mold diseases [39,40,41]. As such, ordering antifungal susceptibility testing is up to the discretion of the ordering clinician, as previously discussed. 

### 7.3. Triazoles

Oral triazole therapy is considered to be the standard of care [8,13]. Oral itraconazole and voriconazole are preferred, based on the body of evidence supporting their use [115,116,117,118,119,120,121]. The highly variable bioavailability of itraconazole limits its reliability [122]. However, a new formulation, SUBA-itraconazole, has improved bioavailability, but unproven efficacy, as it has not been studied for the treatment of CPA [123]. Like other triazoles, itraconazole has been associated with gastrointestinal disturbances and hepatotoxicity, but treatment discontinuation is rarely required [115]. One recent study demonstrated that voriconazole may be more effective than itraconazole [124]. However, voriconazole carries the risk of significant adverse effects, including neurotoxicity, phototoxicity, increased risk of dermatologic malignancies, and periostitis [125,126,127]. The phenomenon of accelerated metabolism (also referred to as autoinduction) that is particular to voriconazole requires attention and regular therapeutic drug monitoring [128]. Posaconazole has been recommended as an alternative agent [129,130,131,132]. The delayed-release tablet formulation of posaconazole has enhanced bioavailability compared to the oral suspension formulation [133,134,135]. Posaconazole has been associated with gastrointestinal disturbance, rash, peripheral neuropathy, headache, and fatigue, sometimes leading to treatment discontinuation [129,130]. While not yet recommended in the guidelines, isavuconazole may be an effective option with a superior safety profile [7,121,136]. Extrapolating from the SECURE trial comparing isavuconazole and voriconazole for primary treatment of invasive aspergillosis, isavuconazole may be non-inferior and better tolerated [137]. Isavuconazole has been associated with hepatotoxicity and neurologically related or skin-related adverse events, but has fewer drug interactions than other mold active triazoles [121,138]. Direct head-to-head comparisons have not been performed to establish a clear superiority of one triazole over the others. The choice of which oral triazole to choose should largely depend on availability, tolerability and toxicities, drug-drug interactions, necessity and feasibility of therapeutic drug monitoring, and total costs, factoring in the need for therapeutic drug monitoring. 

### 7.4. Echinocandins

The echinocandins caspofungin, micafungin, and anidulafungin are alternatives to triazole therapy for the treatment of CPA, in the event of triazole failure, resistance, or intolerance [8,13]. The use of an echinocandin as induction therapy for the first 2 to 4 weeks of treatment followed by an oral triazole was shown to be similarly effective and better tolerated [139]. Caspofungin has been administered in a cyclical pulsed fashion, in combination with oral triazoles between cycles, with mortality rates comparable to triazole-naive patients [140,141]. One study showed no clear difference between micafungin and caspofungin [142]. While there is no published evidence describing outcomes with anidulafungin, there does not appear to be any pharmacological basis to suggest that it would be superior or inferior to the other echinocandins. The echinocandins are generally safe and well-tolerated, but can cause hepatotoxicity and infusion reactions [143]. Their use is limited by their exclusive intravenous route of administration. 

### 7.5. Amphotericin B Products

Amphotericin B products are also alternatives to triazole therapy for the treatment of CPA in the event of triazole failure, resistance, or intolerance [8,13]. Lipid formulations of amphotericin B (amphotericin B lipid complex and liposomal amphotericin B) have improved outcomes and lesser adverse effects compared to conventional amphotericin B deoxycholate [8,143]. One study showed that liposomal amphotericin B led to a clinical response in 73.8% of patients during their first course, but the risk of acute kidney injury was a concern [144]. Amphotericin B deoxycholate does not appear to have a positive impact, and should only be considered when other agents cannot be used [5,145]. 

### 7.6. Combination Antifungal Therapy

There are no guideline recommendations supporting the use of combination antifungal therapy for CPA [8,13]. Extrapolating from studies evaluating combination antifungal therapy for invasive aspergillosis, there does not appear to be any clear proven benefit compared to monotherapy [146,147]. 

### 7.7. Local Direct Antifungal Therapy

The local direct instillation of antifungal agents can be attempted in patients who have failed or intolerant of systemic antifungal therapy, are deemed to not be candidates for surgical resection, and are not at an increased risk for bleeding [13]. The largest amount of published experience is with amphotericin B (paste or solution), although nystatin, miconazole, itraconazole, and sodium iodide have also been used [13,148,149,150,151,152,153,154,155,156]. Antifungal agents are usually administered by a percutaneous catheter placed directly into the cavity, but can also be administered by endobronchial catheterization [13]. 

### 7.8. Novel Antifungal Therapies

A number of novel antifungal agents (e.g., amphotericin B cochleates, ibrexafungerp, nikkomycin Z, olorofim, rezafungin) are in various stages of development [157,158,159,160,161,162]. Their role, if any, in the treatment of chronic pulmonary aspergillosis is yet to be established. 

### 7.9. Immunotherapies

Corticosteroids are a known risk factor for the progression and dissemination of CPA, particularly in the absence of antifungal therapy [8,13,163]. However, patients with other medical conditions, such as sarcoidosis, rheumatoid arthritis, chronic obstructive pulmonary disease, allergic bronchopulmonary aspergillosis, and asthma may require corticosteroids or other immunomodulators to manage their underlying disease. Corticosteroids and other immunomodulators can be cautiously used for symptomatic management if their fungal disease is being adequately treated with antifungal therapy [8,13,164]. Inhaled corticosteroids should be discontinued or minimized in those in whom it is safe and feasible to do so [8]. Interferon gamma has been used as an adjunctive therapy in combination with antifungal therapy in a small number of cases, but it is not routinely recommended [5,165,166]. Other immunotherapies that may have a future role in specific situations include granulocyte colony-stimulating factor, granulocyte macrophage-stimulating factor, granulocyte transfusion, adoptive T-cell therapy, chimeric antigen receptor T-cell therapy, and vaccines [167,168,169]. 

### 7.10. Surgical Therapy

Chronic pulmonary aspergillosis can be complicated by hemoptysis of variable severity. Hemoptysis can be managed with tranexamic acid [8,13,170,171]. Bronchial artery embolization and bronchial occlusion using silicone spigots have also been performed [121,172]. 

Surgery should be considered in all patients with severe hemoptysis, or those who are refractory or intolerant to systemic antifungal therapies [8,13]. Surgical procedures include bullectomy, segmentectomy, sublobar resection, wedge resection, lobectomy, pleurectomy, pneumonectomy [13,173,174,175,176,177,178]. Video-assisted thoracic surgery is generally preferred to open surgery, but is contingent upon the extent of surgery that needs to be performed [179]. The success of surgery largely depends on the ability to completely resect the diseased area, without the spillage of fungal elements into the pleural space [13]. Success rates for surgical resection of chronic pulmonary aspergillosis have been historically suboptimal but have improved in recent years [173,174,175,176,177,178]. 

## 8. Outcomes and Unmet Needs

Treatment responses are slow. Patients who respond usually do so after 4 to 6 months of antifungal therapy, and this should be considered the minimum duration of antifungal therapy [8,13,129]. Those with suboptimal responses may respond after nine months of antifungal therapy [129]. One study showed response rates to posaconazole of 61% at 6 months of therapy and 46% at 12 months of therapy [129]. This was similar to response rates from itraconazole (ranging from 29% to 93%), voriconazole (ranging from 44 to 86%), and micafungin (ranging from 55% to 73%) [129]. Responders may have superior outcomes from lifelong suppressive therapy [8,13,115]. Relapses may occur after treatment discontinuation, but this is not always the case [115,180]. A prospective, randomized controlled trial, comparing six months of itraconazole, plus supportive care versus supportive care alone, found that clinical or radiographic responses were significantly higher in the itraconazole arm, but patients in both groups demonstrated similar rates of clinical and/or radiographic worsening in the post-treatment follow-up period [115]. A retrospective study evaluating 39 patients with CPA found that one-third of cases relapsed after discontinuing azole treatment, with the highest risk occurring in those with multilobar involvement [180]. Late relapses, months or years later, have also been reported, highlighting the need for continued surveillance after treatment completion [5].

Poorer outcomes have been associated with older age, worse dyspnea, lower activity scores, lower body mass index, hypoalbuminemia, the presence of an aspergilloma, prior or current nontuberculous mycobacterial infection, and chronic obstructive pulmonary disease [181,182]. Studies have demonstrated a high initial mortality of 30% over 3–6 months in older patients, 28% mortality at one year, 20–50% mortality at 3 years, and 50–80% mortality at 7–10 years [174,175,176,177,181,182,183,184]. However, one recent study showed substantially improved 1-, 3-, 5-, and 10-year overall survival rates of 100%, 93%, 93%, and 92%, respectively, with the highest relapse rates occurring within the first three years after surgery [178]. 

Reliable and reproducible *in vitro* antifungal susceptibility testing, in addition to studies showing its correlation with clinical outcomes, would aid clinicians in selecting antifungal therapy. Newer antifungal agents that are more effective (allowing shorter durations of curative therapy), better tolerated, have fewer drug-drug interactions, offer multiple routes of administration, and are cheaper, are needed. The utility and safety of combination antifungal therapy should be explored. The role of immunomodulation in the treatment of CPA warrants further research. 

## Figures and Tables

**Table 1:** Diagnostic Criteria for Different Forms of Chronic Pulmonary Aspergillosis [8,13].

Disease	Radiographic	Laboratory	Clinical
Simple aspergilloma	Single cavity containing a fungal ball No radiological progression over at least three months	Serological or microbiological evidence of *Aspergillus* spp.	Minor or no symptoms
*Aspergillus* nodule	One or more nodules +/− cavitation	Serological or microbiological evidence of *Aspergillus* May resemble other infection or neoplasm, definitive diagnosis by histopathology	Minor or no symptoms
Chronic cavitary pulmonary aspergillosis (CCPA)	One or more cavities, possibly containing aspergilloma Overt progression (new cavities, increasing pericavitary infiltrates or pleural thickening) over at least three months	Serological or microbiological evidence of *Aspergillus* spp. *Aspergillus* IgG positive	Significant cough, hemoptysis, fatigue, or weight loss
Chronic fibrosing pulmonary aspergillosis (CFPA)	Fibrotic destruction of at least two lobes complicating CCPA May manifest as consolidation or large cavities with surrounding fibrosis	Serological or microbiological evidence of *Aspergillus* spp. *Aspergillus* IgG positive	Significant pulmonary and systemic complaints Decline in lung function
Subacute invasive aspergillosis (SAIA)	Variable radiographic features: cavitation, nodules, progressive consolidation over 1–3 months	Serological or microbiological evidence of *Aspergillus* spp. Positive *Aspergillus* galactomannan (blood or BAL) Histopathology: hyphae with tissue invasion	Occurs in mildly immunocompromised patients Weight loss, cough, hemoptysis, fever

**Table 2:** Macroscopic and Microscopic Morphological Characteristics of Clinically Relevant *Aspergillus* spp. [24,25]

Species	Macroscopic Morphology	Microscopic Morphology
*A. fumigatus*	Velvety or powdery texture Blue green when young, becoming smokey dark green/gray with age Colony spreading	Smooth conidiophore Uniseriate form Compact, columnar arrangement Phialides on upper two-thirds of vesicle Conidia round and smooth to rough
*A. niger*	Cottony or granular texture White with specks of black and yellow when young, turning completely black when mature Colony spreading	Smooth, large conidiophore Biseriate form Radial arrangement Phialides cover entire vesicle Conidia round and rough
*A. flavus*	Cottony or granular texture White turning yellow-green when mature Colony spreading	Rough, spiny conidiophore Uniseriate and biseriate forms Loose, radial arrangement Phialides cover entire vesicle Conidia round and rough
*A. terreus*	Velvety or powdery texture White with yellowish reverse when young, maturing to cinnamon-brown obverse with yellow diffusing pigment on reverse Colony spreading	Smooth conidiophore Biseriate form Compact, columnar arrangement Phialides on upper half of vesicle Conidia round and smooth
*A. nidulans*	Velvety texture White with orange reverse at first, maturing to dark green with white border with some yellow specks (where cleistothecia form) and deep red reverse Colony spreading	Smooth conidiophore Biseriate form Loose, columnar arrangement Phialides on upper half of vesicle Round Hulle cells and Cleistothecia with red ascospores may be present Conidia round and smooth to rough
*A. versicolor*	Velvety texture Initially white maturing to various colors (tan, green, yellow, beige) with white border with yellow, red, or orange reverse and reddish exudate. Colony bordered and compact	Smooth conidiophore Biseriate form Loose, radial arrangement Phialides cover entire vesicle Round Hulle cells may be present Conidia round and smooth to rough Penicillium-like structures present
*A. sydowii*	Velvety texture Initially white maturing to deep bluish- emerald green with white border Colony bordered and compact	Smooth conidiophore Biseriate form Loose, radial arrangement Phialides cover entire vesicle Conidia round and smooth to rough Penicillium-like structures present
*A. ustus*	Velvety or powdery texture Dull grayish brown with yellow diffusing pigment on reverse Colony spreading	Smooth conidiophore Biseriate form Loose, radial arrangement Phialides on upper half to three-quarters of vesicle Conidia round and rough Irregularly-shaped Hulle cells may be present
